# Identification of miRNAs Involved in Stolon Formation in *Tulipa edulis* by High-Throughput Sequencing

**DOI:** 10.3389/fpls.2016.00852

**Published:** 2016-06-21

**Authors:** Zaibiao Zhu, Yuanyuan Miao, Qiaosheng Guo, Yunhao Zhu, Xiaohua Yang, Yuan Sun

**Affiliations:** ^1^Institute of Chinese Medicinal Materials, Nanjing Agricultural UniversityNanjing, China; ^2^College of Pharmacy, Henan University of Chinese MedicineZhengzhou, China

**Keywords:** *Tulipa edulis*, miRNAs, stolon formation, high-throughput sequencing, gene expression

## Abstract

MicroRNAs (miRNAs) are a class of endogenous, non-coding small RNAs that play an important role in transcriptional and post-transcriptional gene regulation. However, the sequence information and functions of miRNAs are still unexplored in *Tulipa edulis*. In this study, high-throughput sequencing was used to identify small RNAs in stolon formation stages (stage 1, 2, and 3) in *T. edulis*. A total of 12,890,912, 12,182,122, and 12,061,434 clean reads were obtained from stage 1, 2, and 3, respectively. Among the reads, 88 conserved miRNAs and 70 novel miRNAs were identified. Target prediction of 122 miRNAs resulted in 531 potential target genes. Nr, Swiss-Prot, GO, COG, and KEGG annotations revealed that these target genes participate in many biologic and metabolic processes. Moreover, qRT-PCR was performed to analyze the expression levels of the miRNAs and target genes in stolon formation. The results revealed that miRNAs play a key role in *T. edulis* stolon formation.

## Introduction

**Figure 1:**
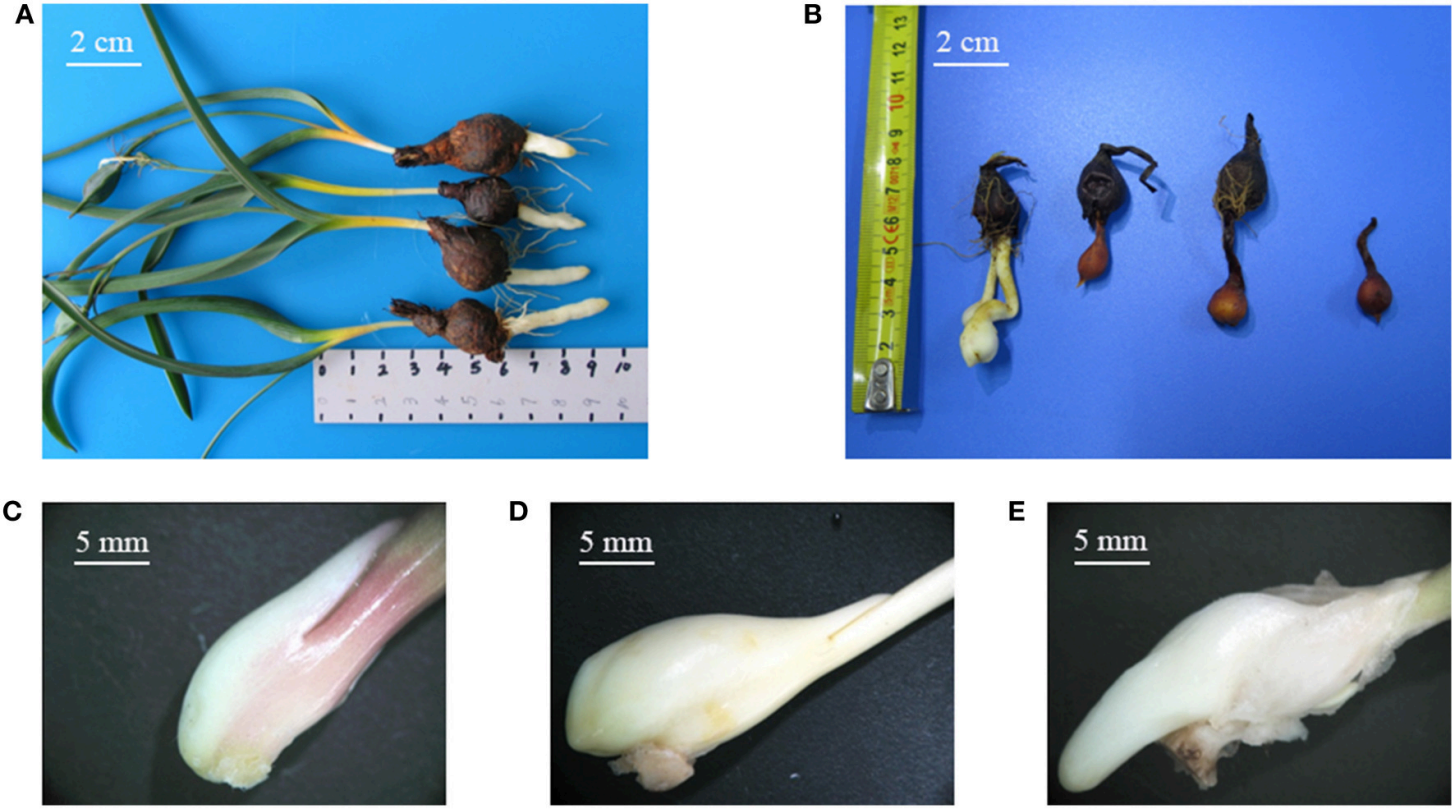
**(A)** *Tulipa edulis* with elongated stolon; **(B)** *T. edulis* stolon developed into a new bulb; **(C–E)** *T. edulis* stolon formation at the initial, middle, and later stages. Scale: **(A,B)** 2 cm; **(C–E)** 5 mm.

*Tulipa edulis* is a perennial herb in the family Liliaceae and is an important medicinal plant in China. The dry bulb of *T. edulis* is commonly used in the treatment of a variety of tumors (Lee et al., 2010; Lin et al., 2015). Currently, *T. edulis* is mainly dependent on wild resources, so there are huge gaps in market demand. The stolon is one of the main asexual reproductive organs of *T. edulis*, with a unique morphology characterized by the absence of an adventitious root, as well as the absence of an obvious node or internode (Figure 1). Determining the mechanism of stolon formation has great significance for the promotion of research into *T. edulis*. In our previous works, we have performed an RNA-seq analysis of the transcriptomes of stolons at different developmental stages. Some differentially expressed genes (DEGs) were detected and annotated to involved in many physiological and biochemical processes, such as material and energy metabolism, hormone signaling, cell growth, and transcription regulation (Miao et al., 2016). However, to explore the molecular mechanism of stolon formation, the research on the characteristics and morphological development of the *T. edulis* stolon is still very limited. MicroRNAs (miRNAs) are noncoding RNAs comprising 21–24 nucleotides (nt) that are processed from long self-complementary precursor RNAs. In general, miRNAs regulate gene expression by completely or partially binding target mRNAs (Obernosterer et al., 2006). Since the first miRNA lin-4 was identified in *Caenorhabditis elegans* (Lee et al., 1993), more and more miRNAs, which play a pivotal role in post-transcriptional gene regulation, have been identified in plants and animals. The latest miRNA database (miRBase version 21.0) contains 28,645 entries representing hairpin pre-miRNAs and 35,828 mature miRNA products from 233 species. However, in plants, most studies of miRNAs have focused on model species. Exploring the miRNAs that are present in non-model species without reference genome data can help to broaden the species database. Recently, high-throughput sequencing has been successfully used to discover species-specific or lowly expressed miRNAs, such as those found in *Aquilaria sinensis* (Gao et al., 2012), *Brachypodium* (Zhang et al., 2009), and olive (Donaire et al., 2011). This powerful tool allows discovery of conserved and novel miRNAs in plant species without whole-genome data.

Many researchers have reported that miRNAs play an important role in various stages of plant development, such as bud differentiation, flowering, leaf growth, and root elongation (Aukerman and Sakai, 2003; Chen, 2004; Wang J. W. et al., 2011). For example, miR160 plays important roles in the regulation of floral organ development and auxin signaling (Wu et al., 2006), whereas miR167 regulates sex differentiation in *Arabidopsis* (Mallory et al., 2005). Some auxin response transcription factors (*ARFs*) have been shown to regulate plant development and are targeted by miR160 and miR167 (Mallory et al., 2005; Wu et al., 2006; Chapman and Estelle, 2009). MiR156/157 and miR172 target *SPL* genes regulating floral transition in *Arabidopsis* (Gandikota et al., 2007; Jung et al., 2007). Furthermore, miR172 can also regulate floral organ identity and flowering time by translational repression or target binding of the *APETELA2* genes (Aukerman and Sakai, 2003; Varkonyi-Gasic et al., 2012).

To date, miRNA expression levels in *T. edulis* have not been reported. To identify the key miRNAs involved in stolon formation in *T. edulis*, we performed high-throughput sequencing and bioinformatics analysis to compare miRNA populations from three libraries of three developmental stages (stage 1, 2, and 3). A total of 88 conserved and 70 novel miRNAs were identified in *T. edulis*, and differentially expressed miRNAs among the three libraries were screened. Furthermore, the potential miRNA-target genes were predicted and annotated by the GO, COG, and KEGG databases. In addition, quantitative real-time PCR (qRT-PCR) was used to analyze the expression patterns of miRNAs and their target genes in the aforementioned three developmental stages. The results serve as a valuable information resource regarding the miRNAs that are associated with stolon formation in *T. edulis*.

## Materials and methods

### Plant materials

The experiment was performed under natural day/night conditions in the greenhouse of the Institute of Chinese Medicinal Materials at Nanjing Agricultural University, Nanjing, P. R. China, from October 2013 to March 2014. Disease-free bulbs of uniform size (approximate 1.0–1.5 g) were selected for planting in polyethylene plastic discs filled with nutrient-enriched soil. Stolon formation was divided into three stages: stage 1, the initial period of stolon formation, 41 days after planting (DAP), Figure 1C; stage 2, the middle period of stolon formation, 118 DAP, Figure 1D; and stage 3, the later period of stolon formation, 133 DAP, Figure 1E. Fertilizer and water conditions were strictly controlled during the experiment. The stolon samples were collected at the three stages, immediately frozen in liquid nitrogen and then stored at −80°C.

### RNA extraction, construction of the small RNA library, and solexa sequencing

Total RNA was extracted from each sample by the modified CTAB method (Chang et al., 1993). The extracted RNAs of each sample were used to construct three small RNA libraries. In brief, polyacrylamide gel electrophoresis was used to enrich small RNA molecules in the range of 18–30 nt, which were ligated to adapters at their 5′- and 3′-ends and converted into cDNA by reverse transcription-PCR (RT-PCR). Three libraries were sequenced with an Illumina Hi-seq 2500 platform (Biomarker Technologies Co., Ltd, Beijing, China).

### Prediction of conserved and novel MiRNAs

After removing the low-quality sequences, sequences with lengths < 16 nt and >30 nt and unique reads of 15–30 nt were used for further analyses. All clean reads were annotated as genome RNAs, rRNAs, scRNAs, snRNAs, snoRNAs, tRNAs according to the Genbank (http://www.ncbi.nlm.nih.gov/genbank) and Rfam databases (http://rfam.sanger.ac.uk). To identify the conserved miRNAs in *T. edulis*, small RNA sequences were used for a BLASTn search against the known mature plant miRNAs in the miRBase (Version 19.0). The unique sequences which have more than 90% sequence similarity (no more than two mismatches) with known plant miRNA family sequence are considered to be conserved miRNAs. The transcriptome data (the NCBI accession number SRR 2177458) were used to predict novel miRNAs.

### Prediction of potential MiRNA target genes and functional annotation of target genes

The potential target genes of the miRNAs were predicted using the psRNA Target program (http://plantgrn.noble.org/psRNATarget/) (Dai and Zhao, 2011). The sequences of miRNAs were submitted in NCBI, under the accession number SRP074213. The functional annotation of potential target genes was performed using diverse protein databases, including Gene Ontology (GO; Ashburner et al., 2000), Cluster of Orthologous Groups (COG; Tatusov et al., 2000) and the Kyoto Encyclopedia of Genes and Genomes (KEGG; Kanehisa et al., 2004).

### Differential expression of MiRNAs between three libraries

To identify the differentially expressed miRNAs, the frequencies of the miRNAs in three libraries were normalized to one million of the total number of miRNA reads in each sample. The differentially expressed miRNAs were identified among the three libraries using IDEG6 software (Romualdi et al., 2003). A threshold of a false discovery rate < 0.01 and a fold-change ≥2 were used to determine significant expression changes. Then the Hochberg Benjamini correction method (Benjamini and Hochberg, 1995) was used for correcting the *p*-value which obtained from the original hypothesis test, finally the false discovery rates were used as a key indicator to select the differentially expressed miRNAs.

### QRT-PCR analysis of MiRNAs and target genes

Total RNAs were extracted from three stolon samples by the modified CTAB method. Then, the small RNAs were reverse-transcribed into cDNA using the One Step PrimeScript^®;^ miRNA cDNA Synthesis Kit (TaKaRa, Dalian, China). qRT-PCR was performed using a SYBR Premix EX Taq Kit (TaKaRa, Dalian, China) in a 20 μl reaction volume containing 2 μl of cDNA, 7 μl of diluted cDNA, 0.5 μl of each primer, and 10 μl of PCR Master Mix. The reactions were carried out using the ABI 7300 Real-Time PCR System (Applied Biosystems, Foster City, CA, USA) according to the manufacturer's instructions. 5S rRNA was used as the reference gene. Three replicates were performed for each sample, and the relative gene expression levels were calculated using the 2^−ΔΔCt^ method (Pfaffl, 2001). The primers used for the miRNAs and target genes are shown in Table S1.

## Results

### High-throughput sequencing analysis of small RNAs

**Figure 2:**
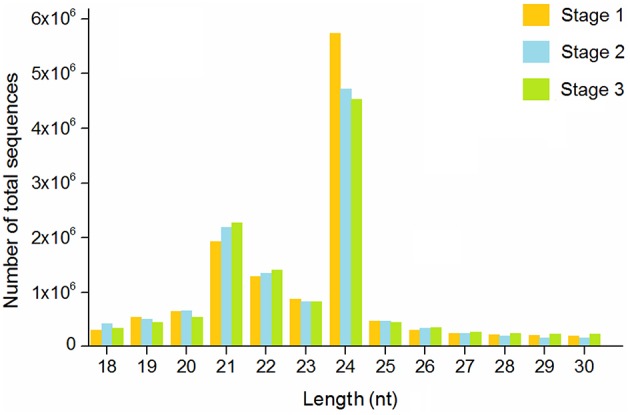
**Length distribution of sequence reads in ***T. edulis*****. Stage 1, *T. edulis* stolon formation at the initial stage; Stage 2, *T. edulis* stolon formation at the middle stage; Stage 3, *T. edulis* stolon formation at the later stage.

To determine the possible involvement of miRNAs in stolon formation, three small RNA libraries were constructed for sequencing. Deep sequencing generated 19,054,308, 18,379,715, and 17,472,455 reads in the three libraries, respectively. After removing low quality reads, adapter sequences, and sequences with lengths shorter than 15 nt or longer than 30 nt, the clean reads were 12,890,912 for stage 1, 12,182,122 for stage 2, and 12,061,434 for stage 3. The length distribution of the small RNAs in the three libraries is shown in Figure 2. The most abundant small RNAs in all of the libraries are 24 nt in length, followed by 21 nt length. A similar result was reported in *Arabidopsis thaliana* and peanut (Rajagopalan et al., 2006; Chi et al., 2011). Previous studies have reported that 21 nt small RNAs have been described as the typical miRNAs and that 24 nt small RNAs involve repeats and heterochromatin formation (Pontes et al., 2009). Reads from all *T. edulis* libraries were annotated according to the GenBank and Rfam databases, and the numbers and proportions of the different categories are given in Table 1. As a result, reads with 11.50%, 13.11%, and 12.97% matches to genome RNAs were identified in stage 1, 2, and 3, respectively. Excluding the unannotated reads, the most abundant class was rRNA, which accounted for almost 15%–20%.

**Table 1:** **Statistics of small RNA sequence reads ***in T. edulis*****.

**Type**	**Stage 1**		**Stage 2**		**Stage 3**	
	**Number**	**Percentage (%)**	**Number**	**Percentage (%)**	**Number**	**Percentage (%)**
Genome RNA	1481965	11.50	1597305	13.11	1564278	12.97
rRNA	2033198	15.77	2344894	19.25	2008866	16.66
scRNA	0	0.00	0	0.00	0	0.00
snRNA	15792	0.12	4752	0.04	6854	0.06
snoRNA	238	0.00	352	0.00	280	0.00
tRNA	321373	2.49	171061	1.40	154487	1.28
Repbase	5491	0.04	10014	0.08	7736	0.06
Other	9032855	70.07	8053744	66.11	8318933	68.97
Clean_reads	12890912	100.00	12182122	100.00	12061434	100.00

*Stage 1, T. edulis stolon formation at the initial stage; Stage 2, T. edulis stolon formation at the middle stage; Stage 3, T. edulis stolon formation at the later stage*.

### Discovery of conserved and novel MiRNAs

To identify the conserved miRNAs in *T. edulis*, small RNA sequences were compared with the currently known mature plant miRNAs in the miRBase. A total of 112,903, 149,433, and 158,159 small RNAs could be matched with previously known miRNAs in the three libraries, which accounted for 0.08%, 0.06%, and 0.04% of all clean reads, respectively. A significant predominant bias of U was observed in the first nucleotides of all putative miRNA sequences across all libraries (Figure S1), which was consistent with the typical miRNA sequence patterns. The miRNAs that perfectly matched with conserved miRNA sequences or had stem-loop precursors were further identified as conserved miRNAs. Finally, a total of 88 small RNAs in *T. edulis* were identified as orthologs of known miRNAs in other species.

To uncover novel miRNAs sequences in *T. edulis*, all unannotated small RNAs were searched against the transcriptome sequence data (SRR 2177458; Miao et al., 2016). After searching for potential miRNA precursors and predicting stem-loop hairpin structures, 70 unique sequences were identified as novel miRNAs in *T. edulis* and renamed ted-miR1 to ted-miR70. The sequences of conserved and novel miRNAs are represented in Tables S2, S3.

### Prediction and functional annotation of MiRNA target genes

MiRNAs are involved in many biological processes via negative regulation of their target genes (Bartel, 2004; Jones-Rhoades et al., 2006). In this study, we searched for putative target genes using TargetFinder software, with default parameters (Fahlgren and Carrington, 2010). A total of 531 potential target genes were predicted in *T. edulis*. Among them, there were 331 and 206 target genes for 68 conserved and 54 novel miRNAs, respectively. The majority of miRNAs had more than one target gene.

**Figure 3:**
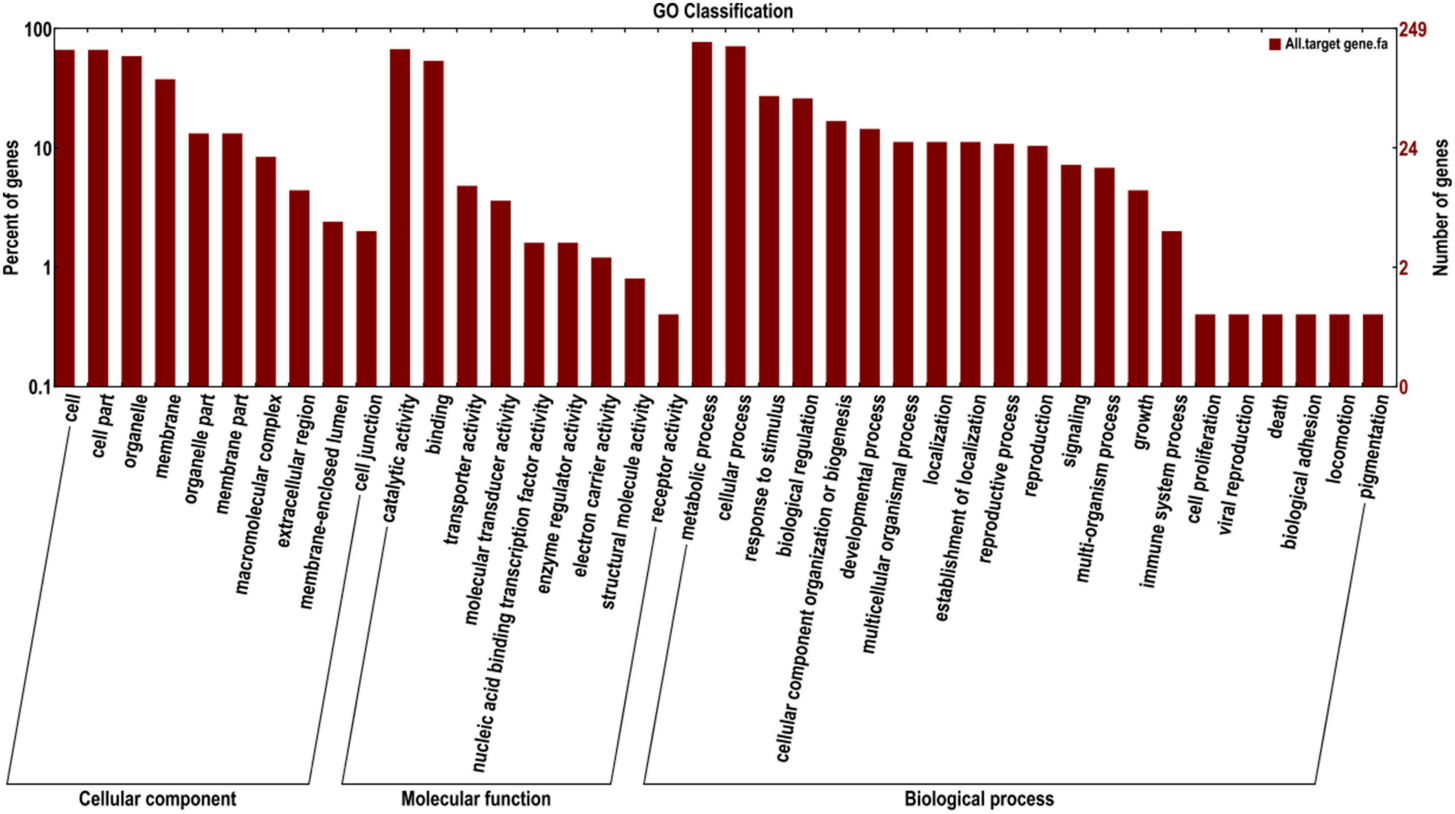
**GO classification assigned to miRNA target genes in ***T. edulis*****.

**Figure 4:**
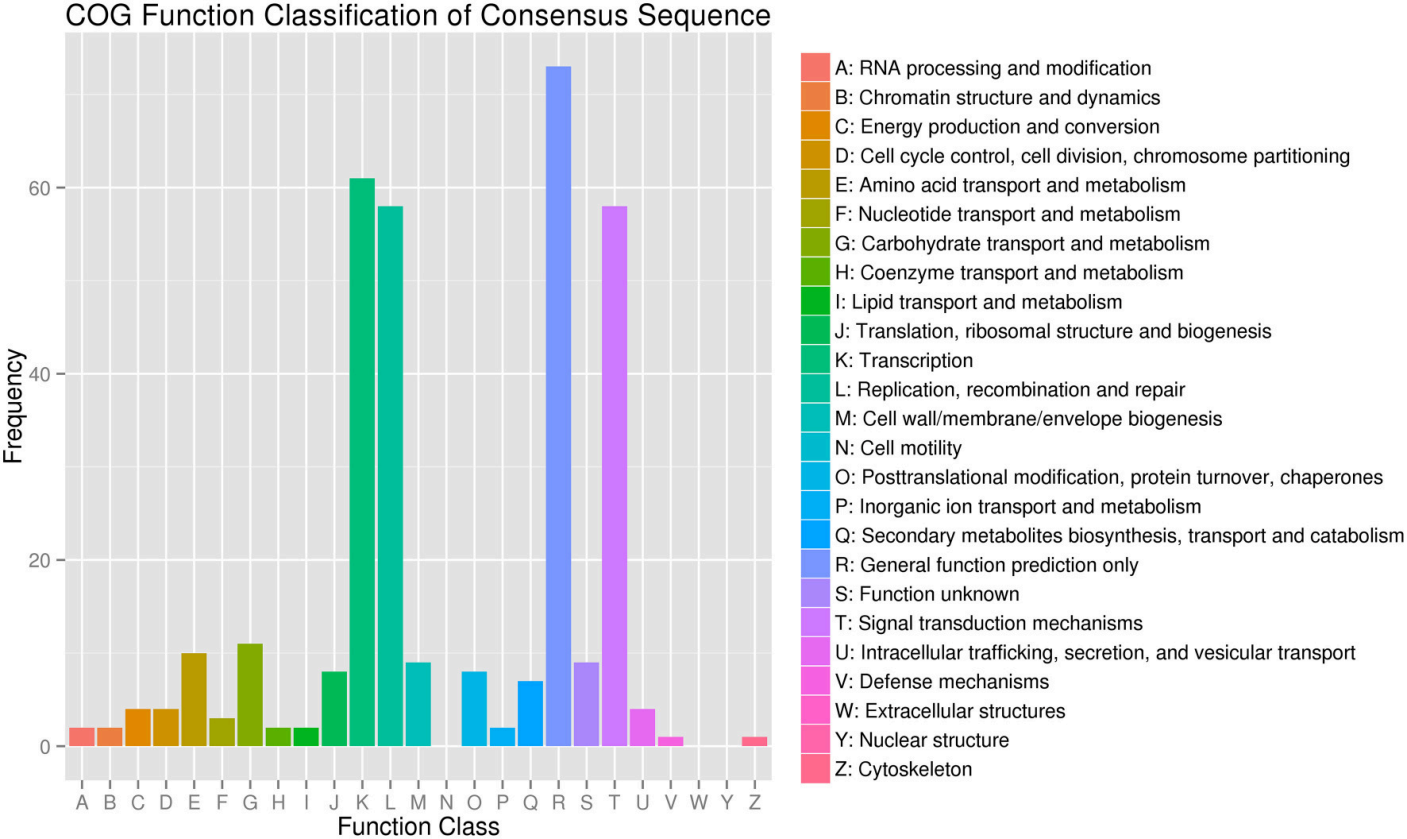
**COG classification of miRNA target genes in ***T. edulis*****.

To better understand the functions of the target genes, five public databases (Nr, Swiss-Prot, GO, COG, and KEGG) were used to annotate gene functions. Among all target genes, 339 target genes had annotation information (Table 2). A total of 335 and 303 target genes were homologous with known proteins in the Nr and Swiss-Prot databases, respectively. According to GO analysis, 249 target genes were classified into three gene ontology categories and 40 functional categories (Figure 3). In the cellular component, the greatest frequencies in the GO terms were “cell,” “cell part,” and “organelle.” “Catalytic activity” and “binding” accounted for a large proportion of molecular functions. According to biological processes, the target genes were concentrated on “metabolic process” and “cellular process.” Figure 4 summarized the categorization of the target genes according to COG analysis. A total of 157 target genes were functionally clustered into 25 classifications. “General function prediction only” accounted for the largest proportion in COG annotation, followed by “transcription,” “replication, recombination, and repair,” and “signal transduction mechanisms.” KEGG pathway analysis was further used to understand the biological functions and pathways of target genes. By mapping the enzyme commission numbers to the pathways, 61 target genes were assigned to 46 pathways, with the most frequently represented pathways including “purine metabolism,” “pyrimidine metabolism,” “pyruvate metabolism,” and “protein processing in endoplasmic reticulum.”

**Table 2:** **Functional annotation of miRNA target genes in ***T. edulis*****.

**Annotated database**	**Annotated number**	**300 ≤ length < 1000 (bp)**	**Length ≥ 1000 (bp)**
Nr	335	88	234
Swiss-prot	303	72	220
COG	157	20	134
GO	249	52	188
KEGG	61	10	51
Total	339	91	234

### Differential expression of MiRNAs during stolon formation

**Figure 5:**
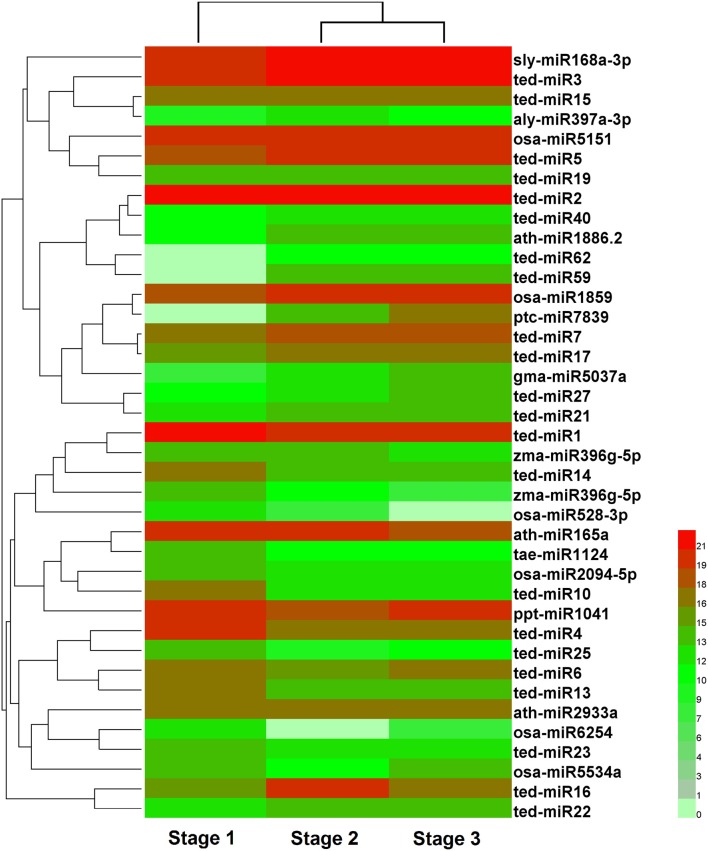
**Cluster analysis of differentially expressed miRNAs in three developmental stages of ***T. edulis*****. Stage 1, *T. edulis* stolon formation at the initial stage; Stage 2, *T. edulis* stolon formation at the middle stage; Stage 3, *T. edulis* stolon formation at the later stage.

To identify the miRNAs involved in stolon formation, the normalized expressions of miRNAs in each sample were compared. IDEG6 software was used to screen the differentially expressed miRNAs. The miRNAs with changes in expression greater than 2-fold in three comparisons are represented in Tables S4–S6. In three comparisons, there were 39, 34, and 10 differentially expressed miRNAs in stage 1 vs. stage 2, stage 1 vs. stage 3, and stage 2 vs. stage 3, respectively. Among them, we found that 22, 19, and 5 differentially expressed miRNAs were novel miRNAs. Compared with stage 1, 21 and 16 miRNAs were up-regulated in stage 2 and stage 3, and 18 and 18 miRNAs were down-regulated in stage 2 and stage 3, respectively. However, in stage 2 vs. stage 3, 5 miRNAs were up-regulated, and 5 miRNAs were down-regulated. This result suggested that most miRNAs are related to stolon formation in the initial period and that novel miRNAs may play important roles in stolon formation. The expression of differentially expressed miRNA in the three libraries was analyzed by hierarchical cluster analysis. The result is shown in Figure 5. A large number of miRNAs showed differential expression during the early stage, but their expression trends during the late stages were similar. At the same time, we observed that the expression levels of most of the miRNAs were higher, indicating that they may play an important role in related biological processes.

### Expression patterns of MiRNAs at different stages of stolon formation

**Figure 6:**
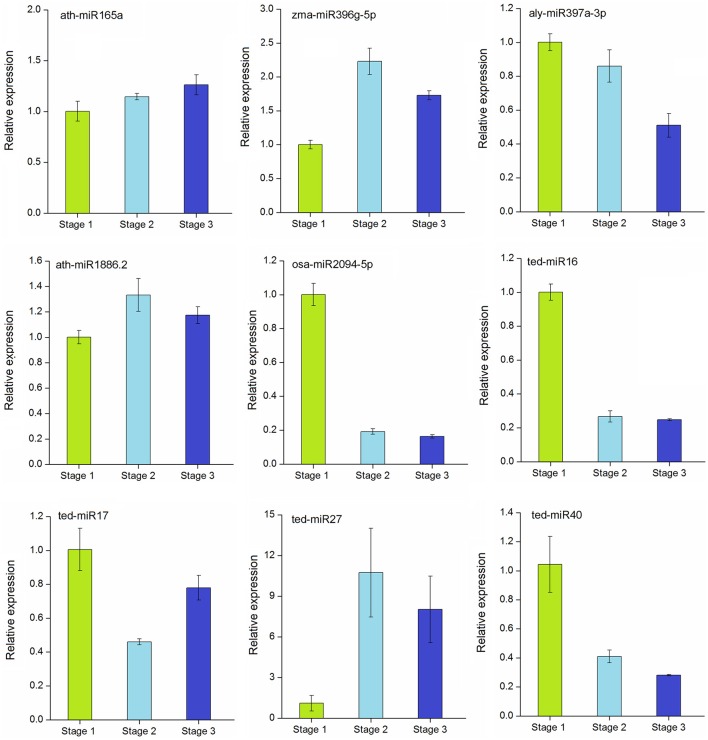
**qRT-PCR validation of selected conserved and novel differentially expressed miRNAs in ***T. edulis*****. Stage 1, *T. edulis* stolon formation at the initial stage; Stage 2, *T. edulis* stolon formation at the middlestage; Stage 3, *T. edulis* stolon formation at the later stage.

To verify the expression profiles obtained from sequencing, the expression levels of five conserved (ath-miR165a, zma-miR396g-5p, aly-miR397a-3p, ath-miR1886.2, and osa-miR2094-5p) and 4 novel (ted-miR16, ted-miR17, ted-miR27, ted-miR40) differentially expressed miRNAs were examined by qRT-PCR, and the stage 1 was used as the control. As shown in Figure 6, the relative expressions of most miRNAs were in agreement with previous sequencing data. Of the conserved miRNAs, the expressions of ath-miR165a and ath-miR1886.2 increased slightly during stolon formation. The relative expression of zma-miR396g-5p was higher in stage 2 and stage 3 than in stage 1. aly-miR397a-3p and osa-miR2094-5p were more highly expressed in stage 1 and were expressed weakly in stage 2 and stage 3. Among the novel miRNAs, ted-miR16 exhibited high expression in stage 1 but showed a large decrease during the stolon developmental stages, whereas the relative expression of ted-miR17 decreased at stage 2 but followed an upward trend thereafter. The relative expression of ted-miR27 increased from stage 1 to stage 3, whereas ted-miR40 expression declined during this period. These observations demonstrated that most of the tested miRNAs were differentially expressed during stolon formation.

### Expression patterns of target genes at different stages of stolon formation

**Figure 7:**
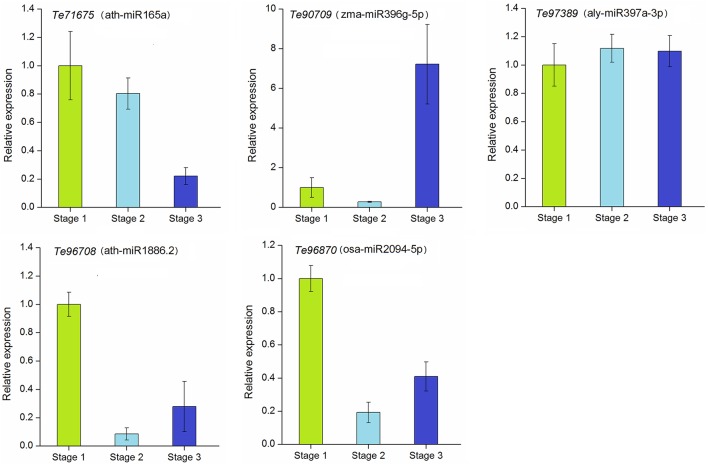
**qRT-PCR analysis of target genes in ***T. edulis*****. Stage 1, *T. edulis* stolon formation at the initial stage; Stage 2, *T. edulis* stolon formation at the middle stage; Stage 3, *T. edulis* stolon formation at the later stage.

To explore the regulation of miRNAs and their target genes in stolon development, we further analyzed the dynamic expression patterns of the corresponding target genes targeted by selected miRNAs. The annotations of the target genes are represented in Table S7. These genes were annotated as transcription factors, biosynthesis-related enzymes, transporter proteins, and putative proteins. As shown in Figure 7, the relative expression levels of these five target genes varied among different developmental stages. Except *Te96708* and *Te97389*, the other three genes showed strong negative correlations with their corresponding miRNAs. The expressions of *Te71675* and *Te96708* were down-regulated during stolon formation as they were targets for the up-regulated ath-miR165a and ath-miR1886.2, respectively. However, the expression of alymiR397a-3p was not significantly correlated with its target gene *Te97389*. Remarkably, *Te96708* and osa-miR2094-5p both experienced decreases in their expression and showed weak expression in stage 2 and stage 3.

## Discussion

The stolon is one of the asexual reproductive organs of *T. edulis*. It has the structural characteristics of both the root and rhizome but also differs significantly from them. Thus, the stolon can serve as experimental material for studying the evolution and environmental adaption of *T. edulis*. Previous studies found that the number of growth years affects stolon formation. Additionally, the environment has a very significant effect on the number of buds and stems produced, especially in moist conditions, which can significantly promote the formation of stolons. Similar conclusions have been reached regarding tulip stolon structure (Le Nard and De Hertogh, 1993; Bach and Ptak, 2005). Current data on the molecular mechanism of stolon formation in *T. edulis* are insufficient. To data, only one RNA-seq analysis of the transcriptomes of *T. edulis* stolons at three developmental stages has been established. Among the three libraries of stolons at three developmental stages, there were 5119 differentially expressed genes (DEGs) which mainly involved in many physiological and biochemical processes (Miao et al., 2016).

MiRNAs regulate a variety of physiological and metabolic processes in plants. A number of studies have revealed that most miRNA-target genes were annotated as transcription factors or functional genes that are directly involved in plant growth and resistance (Sunkar et al., 2007; Rodriguez et al., 2010). To date, there has been no research about miRNA in *T. edulis*. High-throughput technology has been widely applied in the study and functional analysis of miRNAs. In this study, we performed Illumina sequencing and obtained the first miRNA information for *T. edulis*. A total of 12,890,912, 12,182,122, and 12,061,434 clean reads were generated from three libraries. Throughout the sequencing data, reads of 24 nt were dominant in all small RNAs, as has been reported in many other species, such as *Arabidopsis thaliana* and peanut (Rajagopalan et al., 2006; Chi et al., 2011). Based on the sequence conservation and unique structure of mature miRNAs, 88 conserved, and 70 novel miRNAs were identified. However, without the whole-genome data of *T. edulis*, the number of identified miRNAs is relatively small. More miRNAs in *T. edulis* remain to be discovered. Some important and common miRNA families, such as miR169, miR172, and miR156, were detected in our miRNA libraries. It is worth studying further the functions of these miRNAs in *T. edulis*.

Gene regulation is closely related to organ development during plant growth (Dugas and Bartel, 2004; Chen, 2009). High throughput sequencing can help to evaluate the expression levels of miRNAs. Compared with stage 1, 39 and 34 miRNAs were differentially expressed in stage 2 and stage 3, respectively. However, only 10 miRNAs were identified as differentially expressed miRNAs between stage 2 and stage 3, suggesting that most miRNAs play important roles in the initial stage of stolon formation. In addition, in our previous study, we also found that the expression levels of DEGs in stage 1 had a large difference compared to stage 2 and stage 3, while a similar pattern was observed between stage 2 and stage 3 (Miao et al., 2016). That means the initial stage was important in stolon formation. It is worth noting that approximately half of the differentially expressed miRNAs were novel miRNAs, which provided useful information for studying their roles further. Numerous studies have reported that miRNAs play important roles in regulating organogenesis. For example, miR165/miR166 acts as a translational repressor of *HD-ZIP III*, which regulates root and nodule development in *Medicago truncatula* (Boualem et al., 2008). Overexpression of miR165 influences apical meristem formation and vascular development in *Arabidopsis* (Zhou et al., 2007). MiR396 can guide mRNA cleavage of several genes encoding the GRF and bHLH transcription factors that control leaf development (Wang L. et al., 2011; Debernardi et al., 2012). MiR397 is highly expressed in rice seeds and contributes to the regulation of seed development and other agronomic traits (Zhang et al., 2013). In this study, these miRNAs were differentially expressed during stolon formation. Of the miRNAs with significantly different expression levels, the known miRNAs were conserved among plant species. MiR1886 and miR2094 both have been rarely detected in other plants, but in this study, their expressions were specifically altered. This result suggests that some miRNAs may be species-specific. Additionally, the four novel miRNAs showed different expression levels at different stages, which enrich the data for studying the roles of miRNAs.

To explore the functional importance of the miRNAs, target genes were predicted. In the *T. edulis* libraries, 531 target genes were cleaved by 122 miRNAs. Most of the miRNAs targeted several genes. Five public databases were used to annotate the functions of the target genes. Functional annotation results revealed that the majority of the targets are involved in a broad range of physiological and molecular processes, such as cellular and metabolic processes and signal transduction. Previous studies showed that molecular mechanisms, hormone signals, and metabolic pathways are the major intrinsic regulators for controlling plant growth and development (Eveland and Jackson, 2012; Vanstraelen and Benková, 2012). In a previous study, many target genes involved in plant development were successfully identified in potato and cotton (Lakhotia et al., 2014; Xie et al., 2015). The expression patterns of miRNA-target genes in *T. edulis* were also analyzed. As expected, the target genes were negatively correlated with their corresponding miRNAs, which suggested that these miRNAs negatively regulated the participation of their target genes in stolon formation. *Te90709*, which encodes a cysteine proteinase protein, showed a high expression level during the late development stage in *T. edulis* stolon formation. The cysteine proteinase gene is also expressed highly in tomato and sweet potato in leaf development (Drake et al., 1996; Chen et al., 2002). ath-miR1886.2 and aly-miR397a-3p were, respectively predicted to target the transcription factors *Te97389* (belongs to CIPK family) and *Te96708* (belongs to CYP family), and these transcription factors are known to play roles in various aspects of plant growth and development (Morant et al., 2003; Tripathi et al., 2009). Nevertheless, further analysis of these differentially expressed miRNAs and their corresponding target genes are still necessary to explore the miRNA-mediated regulatory mechanisms of stolon formation in *T. edulis*.

## Conclusions

In conclusion, this is the first report to identify conserved and novel miRNAs and their target genes using high throughput sequencing in *T. edulis*. Many conserved and novel miRNAs were significantly differentially expressed in stolon development stages and identified as development related miRNAs. Most of the target genes were annotated to participate in growth and development and signal transduction pathways. The digital expression data of differentially expressed miRNAs were further quantified by qRT-PCR to determine up-or down-regulation in stolon formation. The expression levels of target genes were analyzed to gain a better understanding of their function. These results contribute to our understanding on miRNA-mediated regulatory networks in stolon formation in *T. edulis*.

## Author contributions

QG and ZZ conceived and designed the research. ZZ generated the experimental data, performed the data analysis, and drafted earlier versions of the manuscript. ZZ and YM were involved in the sample collection and revised the manuscript. YZ partially generated the experimental data. XY and YS were involved in the sample collection. All authors read, reviewed and approved the final manuscript.

### Conflict of interest statement

The authors declare that the research was conducted in the absence of any commercial or financial relationships that could be construed as a potential conflict of interest.
