# Supplementary material for: Identification of miRNAs Involved in Stolon Formation in Tulipa edulis by High-Throughput Sequencing
Source: Front Plant Sci. 2016 Jun 21;7:852. doi: 10.3389/fpls.2016.00852 (PMC4914584; doi:10.3389/fpls.2016.00852)
Supplement: Supplementary file 1 [file Table1.DOCX]

**TABLE S1 Primers of miRNAs and target genes used for qRT-PCR validation in *T. edulis*.**

| Genes | Sense primer | Anti-sense primer |
| --- | --- | --- |
| zma-miR396g-5p | GGCATCCCACAGCTTTCTTGAAT | - |
| ath-miR165a | TCGGACCAGGCTTCATTCCC | - |
| aly-miR397a-3p | CACGGCGTTACTGTTGCGGAT | - |
| ath-miR1886.2 | GCCGATGAGATGAGCTCTAGCAAC | - |
| osa-miR2094-5p | AGGCTGCTCGTGGTGGCG | - |
| ted-miR16 | GCCCTTAAATATCACCCTCTTGACG | - |
| ted-miR17 | GCCACATGGTTACTTGAGGTTCAGA | - |
| ted-miR27 | GGCGTTCAACATTTCAATCTCTCTG | - |
| ted-miR40 | AGTGGGTCGTCGGCGGCG | - |
| 5S | GGCTCGGCAACGGATATCTCG | - |
| *Te71675* | CCCTCAATCTTGTTCTTCTTCC | ATGTCTCGGGTTCAACTGGT |
| *Te96708* | TTTGATGAATGCCTTTGAGC | TCACGTTTGACAAGACCCAT |
| *Te86162* | TGTCACCAGTCACCCATTCT | ACTGCTGCGACGATGACTAC |
| *Te90936* | GTTGCTGCATTCGTTTGATT | GAGTAGAGCTCAGGGTTCGC |
| *Te87664* | AGCGTGTGGGTAAAGAGACC | AGCTTGTTGATCTGCCTCCT |
| *Te96540* | ATTTCTCCCGGAAAGACACA | CCAATATCTTGTCCCAGGGT |
| *Te86162* | TGTCACCAGTCACCCATTCT | ACTGCTGCGACGATGACTAC |
| *Te97389* | TCAGGTCTTCTTGCTGGCTA | AGAACCACGAAAGTGAGCCT |
| *Te96708* | TTTGATGAATGCCTTTGAGC | TCACGTTTGACAAGACCCAT |
| *actin* | TGTATGTTGCCATTCAGGCT | ATCACCAGAATCCAGCACAA |
