# Supplementary material for: Identification of miRNAs Involved in Stolon Formation in Tulipa edulis by High-Throughput Sequencing
Source: Front Plant Sci. 2016 Jun 21;7:852. doi: 10.3389/fpls.2016.00852 (PMC4914584; doi:10.3389/fpls.2016.00852)
Supplement: Supplementary file 2 [file Table2.DOCX]

**TABLE S2 The sequences of identified conserved miRNAs in *T. edulis*.**

| Conserved miRNAs | Length (nt) | Sequence |
| --- | --- | --- |
| ath-miR165a | 21 | UCGGACCAGGCUUCAUUCCCC |
| ath-miR319a | 21 | UUGGACUGAAGGGAGCUCCCU |
| ath-miR165a | 21 | UCGGACCAGGCUUCAUACCUC |
| sly-miR168a-3p | 21 | CCUGCCUUGCAUCAACUGAAU |
| ppt-miR1041 | 21 | UUUUCGGGUGAUUUGAGGUGG |
| mtr-miR2624 | 22 | UGAAAGACCCCGGUCUACCAUC |
| ptc-miR1450 | 21 | UUCAAUGGCAAUUUGGAUCCU |
| osa-miR5151 | 21 | GAAUGAUGGACUACUCUGGUG |
| osa-miR1859 | 22 | GUUCCUAUGUCACUCAGUUCGG |
| ath-miR2933a | 21 | CAAAUCGGCAACUUUGACGUG |
| ath-miR399a | 21 | UGCCAAAGGAGAAUUGCCCUG |
| zma-miR395c-5p | 20 | CUUCCCUGAAAUGAGCUCCA |
| mtr-miR7700-5p | 20 | UUGGAGUGAAGGGAGCUCCA |
| sbi-miR6221-5p | 21 | CUCUGACUCCGUUGGGAUCCA |
| zma-miR396g-5p | 19 | UCCCACAGCUUUCUUGAAU |
| cre-miR1165-3p | 19 | GCGGACCGCUCGAGCUCCU |
| zma-miR396g-3p | 21 | CUUCAAGAUGUUGAGAACGUG |
| osa-miR5534a | 21 | UGACGACAGGCUCGGACACGC |
| tae-miR1124 | 24 | ACAGGACGACUGACGAUGGAACGU |
| ppt-miR408b | 21 | UGCACUGCCUCUUCCCUGGCU |
| zma-miR396g-5p | 21 | UCCCACAGCUUUAUUGAACUG |
| osa-miR2094-5p | 24 | AGGCUGCUCGUGGUGGCGAACCGG |
| osa-miR6254 | 24 | AGCUCCGGUCGGAUUGGUGGUCGG |
| gma-miR5037a | 21 | ACCUCAAAUCACCCGAAAAGC |
| ppt-miR1041 | 21 | UUUUCGGGGGAUUUGAGGUGG |
| ppt-miR1074 | 20 | AGGGUUGUCAAAGAAAGGAA |
| ath-miR159a | 21 | UUUGGAUUUGCUUGAUGUUUG |
| osa-miR528-3p | 21 | ACUGUGCUCCUCUGAUUUCAG |
| osa-miR5079a | 20 | UUUGGAUCGGAGGGAGCUCG |
| osa-miR5079a | 20 | UUUGGAUCGGAGGGAGCUCG |
| ath-miR1886.2 | 19 | UGAGAUGAGCUCUAGCAAC |
| zma-miR398b-5p | 18 | GGGGCGGAUCUUGGUGGC |
| ptc-miR6427-3p | 24 | GUGGGAAUGGAUGUCUGCCUCGUU |
| bdi-miR7743-3p | 24 | CUUGAACUCCAUCUGUAGGUUUUG |
| hvu-miR6184 | 21 | CGGCGUCGUCCGAGGAGGUGG |
| bdi-miR5165-5p | 24 | AUCUUGGGGCUCUGGAGGCCCUCC |
| hvu-miR6184 | 23 | CGGCGUCGGGGACGAGGGCUUCG |
| zma-miR482-5p | 18 | UGGGAGAUGAGGAUGAAA |
| aly-miR869-3p | 21 | CCUGGGUUUCAGGACCUGCCC |
| mtr-miR169d-3p | 19 | AGCAGGUCGUUGUAGUAUU |
| gma-miR4357 | 23 | UAGUCGUGACUGUGUUGCAAUUG |
| ppt-miR1212 | 20 | GGUGGGACGGUGAGGCUGCU |
| ath-miR399a | 21 | CGCCAAAGCUGUAGACAUCGG |
| hvu-miR6180 | 18 | CGGGUGGAGACGUCGACG |
| bdi-miR7775-5p | 21 | ACCGGUUUGGUCCGUUAACUU |
| cpa-miR8153 | 23 | CGCACUGUGCCUUUUGUUUCAAA |
| aly-miR397a-3p | 21 | ACGGCGUUACUGUUGCGGAUA |
| gma-miR4412-5p | 21 | UGUUGCGGCGGCGCAGUCUCC |
| mtr-miR5753 | 25 | CUUUUGAUCUGCCUGUUGAGUGGGA |
| pta-miR1312 | 25 | CUUGGAGAAGCGCAAAGAGAGGGUG |
| lja-miR397 | 22 | GAUUGAGUUGGCUAACAGAUGC |
| bna-miR6036 | 19 | AUAGUACUCGCCACUGAUG |
| ptc-miR6443 | 23 | GUAUGAUCAGAGCGUUGAGCUAU |
| bdi-miR5182 | 25 | UGAUGAUCUUCGUGUUUACCUCGAG |
| sbi-miR169d-3p | 23 | CGGCGGUCGGACCGGUAGCCGGA |
| gma-miR4374b | 20 | UACUUUCAUGAACUUUGGUU |
| aly-miR162a-5p | 18 | GGAGGCAGAUAGAAGAGC |
| vvi-miR399f | 21 | CGCCGAAGCUGUAGACGUCGG |
| sbi-miR6229-3p | 20 | UUUUUUCUUUUUUGUAGAUU |
| ath-miR842 | 20 | GCAUGGUCUCCAAGAUGGCA |
| ptc-miR6463 | 18 | UGGAUGAUAUAUUUGACU |
| gma-miR5041 | 22 | UUUCAUCUUUAGGCGAGUAGUU |
| osa-miR5157a-5p | 25 | AACUUUUUAAGGAGGCAUUCUGCAG |
| mtr-miR5233 | 24 | GAGGAGGAGGUGGCGUCGGUGAUG |
| mtr-miR5298a | 21 | CGGAUAUGUCUUCAUAAAUGU |
| ppt-miR1212 | 20 | CGUGGGACCUGUAUGCGAGU |
| bdi-miR5181c-5p | 22 | CCUCCGGUCGCGAUUGGUAGUU |
| mtr-miR5558-3p | 21 | AAGAUUUAUAUACUCCUGAGU |
| hvu-miR6211 | 18 | GAGAUCAAUGAGAAUUCA |
| ptc-miR6457b | 21 | UUAGUUUGAUGAUGAUGAUGA |
| ghr-miR7495a | 21 | AUACUUUAUGAAUUCUGACAA |
| bdi-miR7717b-5p | 21 | UCUAAGACCUUCUUGGUCACG |
| osa-miR5160 | 20 | CGAGAUCGGAUCGGGAUCGA |
| ptc-miR7839 | 18 | UGUGGCAUUCUUGAUUUU |
| aly-miR169d-3p | 24 | UCAAGUUGGUUUGCUGAUCAAGUU |
| bdi-miR7766-3p | 22 | AGAGGCUGUGCUGAACUUUACC |
| mtr-miR5286a | 20 | CAGGACAAACUCAGAUGGCU |
| lja-miR7522 | 22 | CACUGCGGAUUGGUUAACCCUU |
| pta-miR948 | 20 | ACAGGCUGCAUCGCAAGAAU |
| bdi-miR5181c-5p | 18 | CCUCCGGUGGGCUCUUGG |
| osa-miR5814 | 20 | GAUCAAGUGAUGAAAGCGUC |
| osa-miR1877 | 23 | AGAUGACAGCAGUGCUACGAACG |
| sbi-miR6221-5p | 20 | UUCUGACUUCUGGAAAAGAU |
| csi-miR3950 | 21 | AUUUUCGGACUAGCUGCAGAC |
| osa-miR5074 | 18 | AAAGGCCAGGACUAGGUU |
| aly-miR3445-5p.2 | 23 | CGUCUUUCCUACUGGAACCCGCU |
| mtr-miR5285a | 21 | UGGGACUUGAAAGCAAGCAGG |
| aly-miR4246 | 23 | CAAUCCAAGCUGAUGAACAGAAG |
