# Supplementary material for: Identification of miRNAs Involved in Stolon Formation in Tulipa edulis by High-Throughput Sequencing
Source: Front Plant Sci. 2016 Jun 21;7:852. doi: 10.3389/fpls.2016.00852 (PMC4914584; doi:10.3389/fpls.2016.00852)
Supplement: Supplementary file 3 [file Table3.DOCX]

**TABLE S3 The sequences of identified novel miRNAs in *T. edulis*.**

| Novel miRNAs | Length (nt) | Sequence |
| --- | --- | --- |
| ted-miR1 | 22 | UUUGACGGCCGGACUGUGUAUU |
| ted-miR2 | 22 | UUCCCUAUUCCGCCCAUUCCGA |
| ted-miR3 | 21 | UAAGCUCCAGCAACACCACGC |
| ted-miR4 | 22 | UUAAUAGUCCGGUCUCCUGACG |
| ted-miR5 | 21 | CUCGACGAGGAUGAGCGGAUA |
| ted-miR6 | 21 | GGCGGCUCAUAGGAAAAGGGC |
| ted-miR7 | 21 | CCUCUACAAUGUCGGACGUGU |
| ted-miR8 | 21 | UUUGAAGAGCUCCUCAAAUGG |
| ted-miR9 | 21 | UUUGAAGAGCUCCUCAAAUGG |
| ted-miR10 | 21 | GCCGAUGCCCGGGCUGAGCGA |
| ted-miR11 | 22 | UUAUCUAGCUCAUCUUCCUUCC |
| ted-miR12 | 21 | UUAAAUGUUUUGCAGACACUC |
| ted-miR13 | 21 | CGUGCGCCGGACCUACGUGGC |
| ted-miR14 | 21 | CGUUUCGGAUCGUAUCGGCCG |
| ted-miR15 | 18 | GUCUCGGUAGAACCUCCA |
| ted-miR16 | 22 | UUAAAUAUCACCCUCUUGACGU |
| ted-miR17 | 21 | CAUGGUUACUUGAGGUUCAGA |
| ted-miR18 | 22 | GGGGCAGGUAAUCGGUGGCAGC |
| ted-miR19 | 21 | UCAAGGCAUGUGAGGCGUAAG |
| ted-miR20 | 21 | GAGGCUGUAGAUGUCAAUCGG |
| ted-miR21 | 22 | UCAAGGCAUGUGAGGCUUAAGC |
| ted-miR22 | 21 | CGUACGAGCUCUGGUACUGAG |
| ted-miR23 | 22 | GAAAGGUGUGGACAUGUCCGAU |
| ted-miR24 | 21 | UGGUACGGAUUUUGACGGAUC |
| ted-miR25 | 24 | AAACGAGCCGAGCUUGAACACAAA |
| ted-miR26 | 21 | UUUCUUUAGGUUACUCUGAUU |
| ted-miR27 | 21 | UCAACAUUUCAAUCUCUCUGU |
| ted-miR28 | 20 | GAUGUCUACAGCUUCGGCGU |
| ted-miR29 | 22 | CCGGUUACUUUGAAAGCUCCCA |
| ted-miR30 | 19 | CUCGGGGUGCGGAUCGUUG |
| ted-miR31 | 20 | CCGAAUCCUGUCAUGAUGCA |
| ted-miR32 | 18 | CGGCGGACGGAUCGAGCU |
| ted-miR33 | 18 | CGGCGGACGGAUCGAGCU |
| ted-miR34 | 23 | GAGAGUGGUGAACUGGAGCUGCA |
| ted-miR35 | 21 | UUCACGUCAAAGUUGCCGAUU |
| ted-miR36 | 23 | AAUUUCUUCGGUGCUGGGAGAUU |
| ted-miR37 | 20 | GAUCCCGAUCUCGACCCCAG |
| ted-miR38 | 25 | CUUGGGUACUGUCAGGGCAUGAGUG |
| ted-miR39 | 21 | UCAAUUCUGUUGCUGAACUGG |
| ted-miR40 | 23 | AGUGGGUCGUCGGCGGCGGCGGC |
| ted-miR41 | 21 | GUGCAGAGAUUGAUGGCUGUC |
| ted-miR42 | 24 | CUCCUCUCUCUCUCUCUCUCUCCU |
| ted-miR43 | 20 | UCGCCUUCAUCAUCGUCGUU |
| ted-miR44 | 24 | UCCGGGCCUUCUACAACGAGAUCA |
| ted-miR45 | 24 | GAACCCGGUGAUCUUGAAUUCAGA |
| ted-miR46 | 21 | AGCGAUGUACCAGAACCCGGC |
| ted-miR47 | 21 | UUACACGUUCGGAUGUGUUCA |
| ted-miR48 | 23 | AUAUAUAUAGAGAGAGAGAGGGU |
| ted-miR49 | 20 | CGGCUGCAGAGUCUGCCGCA |
| ted-miR50 | 20 | CAUCCGCGACUGCAUCGAGA |
| ted-miR51 | 22 | UAGGUCUAAUAAAACAGUUGCA |
| ted-miR52 | 21 | UUCUGGAGGUUAUCAGGCUCC |
| ted-miR53 | 22 | AGGUUCUGGCCAGAGAGGUUCG |
| ted-miR54 | 22 | UGCGGUGCUCGAUCAGCUGAAG |
| ted-miR55 | 25 | AUGCCGGUCAACCAGAAGAGAGGCG |
| ted-miR56 | 24 | GUUGAUGCUGGUGCUUUGCCUCUG |
| ted-miR57 | 25 | CCGGUGUCGGGCAGCAUCAGAUGUG |
| ted-miR58 | 21 | CUAGAAUUCACCCAACCCAAC |
| ted-miR59 | 21 | UUGAUCCAUCACCACCAGAUU |
| ted-miR60 | 21 | UCCGUAUGUGGCACAAAAGUC |
| ted-miR61 | 22 | UCAUCUUGAGGUGGCCUACUCU |
| ted-miR62 | 22 | UGGACUAGUAUUGUAUGGUAUU |
| ted-miR63 | 21 | UCCACCUCGGUCGCCCUCGCC |
| ted-miR64 | 24 | CCGCCAAGGCCGAGGAUGGUGAGA |
| ted-miR65 | 21 | UUGAUAUUGUUGUUGAUUGUU |
| ted-miR66 | 25 | UCAGCCAGCGGACUGGGCUCCUUGU |
| ted-miR67 | 19 | ACAAGGAGGUAUCAGUGCU |
| ted-miR68 | 25 | CGCCCCGGAGUCGAUCGCGCUGCUG |
| ted-miR69 | 21 | GUUUCGGAUCGGGUACGGAUU |
| ted-miR70 | 21 | UCGGAUCUGGCUUAGGAGAUU |
