# Supplementary material for: Identification of miRNAs Involved in Stolon Formation in Tulipa edulis by High-Throughput Sequencing
Source: Front Plant Sci. 2016 Jun 21;7:852. doi: 10.3389/fpls.2016.00852 (PMC4914584; doi:10.3389/fpls.2016.00852)
Supplement: Supplementary file 4 [file Table4.DOCX]

**TABLE S4 The expression patterns of differentially expressed miRNAs between stage 1 and stage 2 during *T. edulis* stolon formation.**

| miRNAs | Stage 1 | Stage 2 | FDR | log_2_FC | regulated |
| --- | --- | --- | --- | --- | --- |
| tae-miR1124 | 16438.87 | 3903.623 | 0.00000 | -2.07423 | down |
| osa-miR5151 | 596719.8 | 1272838 | 0.00000 | 1.092924 | up |
| ath-miR2933a | 225000.1 | 82705.32 | 0.00000 | -1.44387 | down |
| sly-miR168a-3p | 1075348 | 2916993 | 0.00000 | 1.439678 | up |
| ptc-miR7839 | 0 | 23621.92 | 0.00000 | 37.78134 | up |
| osa-miR1859 | 359520.2 | 969299.5 | 0.00000 | 1.43087 | up |
| ath-miR165a | 1567396 | 523342.8 | 0.00000 | -1.58254 | down |
| ppt-miR1041 | 1022565 | 492113.8 | 0.00000 | -1.05513 | down |
| ted-miR1 | 5052925 | 1567455 | 0.00000 | -1.6887 | down |
| ted-miR14 | 92594.46 | 22306.41 | 0.00000 | -2.05347 | down |
| ted-miR3 | 1061034 | 2909443 | 0.00000 | 1.455272 | up |
| ted-miR5 | 500994.2 | 1163708 | 0.00000 | 1.215864 | up |
| ted-miR2 | 2497854 | 6418866 | 0.00000 | 1.361629 | up |
| ted-miR7 | 146272.4 | 313662.5 | 0.00000 | 1.100555 | up |
| ted-miR19 | 8051.692 | 28140.4 | 0.00000 | 1.805279 | up |
| ted-miR15 | 88195.85 | 240222.9 | 0.00000 | 1.445591 | up |
| ted-miR6 | 157902.6 | 58339.85 | 0.00000 | -1.43648 | down |
| ted-miR4 | 641329.5 | 183115.4 | 0.00000 | -1.80831 | down |
| ted-miR59 | 0 | 15099.73 | 0.00000 | 37.13573 | up |
| ted-miR16 | 48676.14 | 607327.2 | 0.00000 | 3.641187 | up |
| ted-miR17 | 46968.2 | 131436.3 | 0.00000 | 1.484607 | up |
| ted-miR25 | 11350.65 | 600.5573 | 0.00000 | -4.24033 | down |
| ted-miR10 | 97514.93 | 6520.337 | 0.00000 | -3.9026 | down |
| ted-miR13 | 67544.75 | 9608.917 | 0.00000 | -2.8134 | down |
| osa-miR5534a | 11630.22 | 1715.878 | 0.00001 | -2.76086 | down |
| zma-miR396g-5p | 24720.11 | 9482.484 | 0.00005 | -1.38235 | down |
| osa-miR6254 | 4696.82 | 0 | 0.00008 | -35.451 | down |
| ath-miR1886.2 | 2966.413 | 13654.78 | 0.00008 | 2.202614 | up |
| zma-miR396g-5p | 9840.957 | 2059.054 | 0.00022 | -2.25682 | down |
| gma-miR5037a | 447.3162 | 6177.161 | 0.00049 | 3.787577 | up |
| osa-miR2094-5p | 12916.26 | 4804.459 | 0.00066 | -1.42674 | down |
| ted-miR40 | 1633.677 | 7206.688 | 0.00197 | 2.141214 | up |
| osa-miR528-3p | 4473.162 | 343.1756 | 0.00222 | -3.70428 | down |
| ted-miR27 | 1341.949 | 6863.512 | 0.00240 | 2.354618 | up |
| aly-miR397a-3p | 894.6324 | 5833.985 | 0.00288 | 2.705115 | up |
| ted-miR22 | 6709.743 | 14413.38 | 0.00448 | 1.103079 | up |
| ted-miR21 | 6404.755 | 13758.22 | 0.00448 | 1.103079 | up |
| ted-miR62 | 0 | 3275.767 | 0.00438 | 34.93111 | up |
| ted-miR23 | 10247.61 | 4258.497 | 0.00556 | -1.26687 | down |
