# Supplementary material for: Identification of miRNAs Involved in Stolon Formation in Tulipa edulis by High-Throughput Sequencing
Source: Front Plant Sci. 2016 Jun 21;7:852. doi: 10.3389/fpls.2016.00852 (PMC4914584; doi:10.3389/fpls.2016.00852)
Supplement: Supplementary file 5 [file Table5.DOCX]

**TABLE S5 The expression patterns of differentially expressed miRNAs between stage 1 and stage 3 during *T. edulis* stolon formation.**

| miRNAs | Stage 1 | Stage 3 | FDR | log_2_FC | regulated |
| --- | --- | --- | --- | --- | --- |
| tae-miR1124 | 16438.87 | 2849.451 | 0.00000 | -2.52836 | down |
| ath-miR165a | 17863126 | 8751234 | 0.00000 | -1.02943 | down |
| sly-miR168a-3p | 1075348 | 3150027 | 0.00000 | 1.55056 | up |
| zma-miR396g-5p | 9840.957 | 325.6515 | 0.00000 | -4.9174 | down |
| ptc-miR7839 | 0 | 133734.2 | 0.00000 | 40.28251 | up |
| zma-miR396g-5p | 24720.11 | 4319.168 | 0.00000 | -2.51686 | down |
| osa-miR1859 | 359520.2 | 1156670 | 0.00000 | 1.685832 | up |
| ath-miR165a | 1567396 | 400877 | 0.00000 | -1.96714 | down |
| gma-miR5037a | 447.3162 | 10420.85 | 0.00000 | 4.542034 | up |
| ted-miR1 | 5052925 | 565434.7 | 0.00000 | -3.15969 | down |
| ted-miR14 | 92594.46 | 9118.243 | 0.00000 | -3.3441 | down |
| ted-miR2 | 2497854 | 6065600 | 0.00000 | 1.279961 | up |
| ted-miR7 | 146272.4 | 352029.3 | 0.00000 | 1.267038 | up |
| ted-miR18 | 131938 | 61548.14 | 0.00000 | -1.10007 | down |
| ted-miR4 | 641329.5 | 257694 | 0.00000 | -1.31541 | down |
| ted-miR59 | 0 | 9769.546 | 0.00000 | 36.50757 | up |
| ted-miR16 | 48676.14 | 124961.4 | 0.00000 | 1.360196 | up |
| ted-miR12 | 67097.43 | 26703.43 | 0.00000 | -1.32923 | down |
| ted-miR17 | 46968.2 | 153707.5 | 0.00000 | 1.710432 | up |
| ted-miR25 | 11350.65 | 1424.726 | 0.00000 | -2.99402 | down |
| ted-miR10 | 97514.93 | 5210.425 | 0.00000 | -4.22615 | down |
| ted-miR13 | 67544.75 | 21167.35 | 0.00000 | -1.674 | down |
| ted-miR27 | 1341.949 | 12049.11 | 0.00000 | 3.166525 | up |
| ted-miR21 | 6404.755 | 18029.25 | 0.00010 | 1.493124 | up |
| ted-miR19 | 8051.692 | 20190.4 | 0.00019 | 1.326305 | up |
| osa-miR2094-5p | 12916.26 | 4274.177 | 0.00019 | -1.59547 | down |
| osa-miR528-3p | 4473.162 | 0 | 0.00028 | -35.3806 | down |
| ath-miR1886.2 | 2966.413 | 12237.64 | 0.00028 | 2.044534 | up |
| osa-miR6254 | 4696.82 | 284.9451 | 0.00035 | -4.04293 | down |
| bdi-miR5165-5p | 3522.615 | 0 | 0.00061 | -35.0359 | down |
| ted-miR20 | 12077.54 | 4559.122 | 0.00173 | -1.4055 | down |
| ted-miR40 | 1633.677 | 6541.348 | 0.00364 | 2.001466 | up |
| gma-miR4412-5p | 447.3162 | 4559.122 | 0.00380 | 3.349389 | up |
| ted-miR51 | 426.9837 | 3730.19 | 0.00908 | 3.126996 | up |
