# Supplementary material for: Identification of miRNAs Involved in Stolon Formation in Tulipa edulis by High-Throughput Sequencing
Source: Front Plant Sci. 2016 Jun 21;7:852. doi: 10.3389/fpls.2016.00852 (PMC4914584; doi:10.3389/fpls.2016.00852)
Supplement: Supplementary file 6 [file Table6.DOCX]

**TABLE S6 The expression patterns of differentially expressed miRNAs between stage 2 and stage 3 during *T. edulis* stolon formation.**

| miRNAs | Stage 2 | Stage 3 | FDR | log_2_FC | regulated |
| --- | --- | --- | --- | --- | --- |
| ptc-miR7839 | 23621.92 | 133734.2 | 0.00000 | 2.501171 | up |
| ted-miR1 | 1567455 | 565434.7 | 0.00000 | -1.47099 | down |
| ted-miR16 | 607327.2 | 124961.4 | 0.00000 | -2.28099 | down |
| ted-miR14 | 22306.41 | 9118.243 | 0.00006 | -1.29063 | down |
| osa-miR5534a | 1715.878 | 9443.895 | 0.00010 | 2.460435 | up |
| cre-miR1165-3p | 28447.45 | 13317.43 | 0.00012 | -1.09498 | down |
| ted-miR13 | 9608.917 | 21167.35 | 0.00036 | 1.139395 | up |
| zma-miR398b-5p | 6806.316 | 759.8536 | 0.00064 | -3.16308 | down |
| gma-miR4412-5p | 343.1756 | 4559.122 | 0.00166 | 3.731737 | up |
| ted-miR29 | 2948.191 | 8392.928 | 0.00470 | 1.509345 | up |
