# Supplementary material for: Identification of miRNAs Involved in Stolon Formation in Tulipa edulis by High-Throughput Sequencing
Source: Front Plant Sci. 2016 Jun 21;7:852. doi: 10.3389/fpls.2016.00852 (PMC4914584; doi:10.3389/fpls.2016.00852)
Supplement: Supplementary file 7 [file Table7.DOCX]

**TABLE S7 The annotation of target genes of the corresponding miRNA in *T. edulis*.**

| MicroRNA | Target gene | Annotation of target gene |
| --- | --- | --- |
| ath-miR165a | *Te71675* | Putative protein |
| zma-miR396g-5p | *Te90709* | Cysteine proteinase RD21a |
| aly-miR397a-3p | *Te97389* | Cytochrome P450 |
| ath-miR1886.2 | *Te96708* | CBL-interacting protein kinase 23 (CIPK23) |
| osa-miR2094-5p | *Te96870* | Alpha-trehalose-phosphate synthase |
