# Supplementary material for: Identification of miRNAs Involved in Stolon Formation in Tulipa edulis by High-Throughput Sequencing
Source: Front Plant Sci. 2016 Jun 21;7:852. doi: 10.3389/fpls.2016.00852 (PMC4914584; doi:10.3389/fpls.2016.00852)
Supplement: Supplementary file 8 [file Image1.PDF]

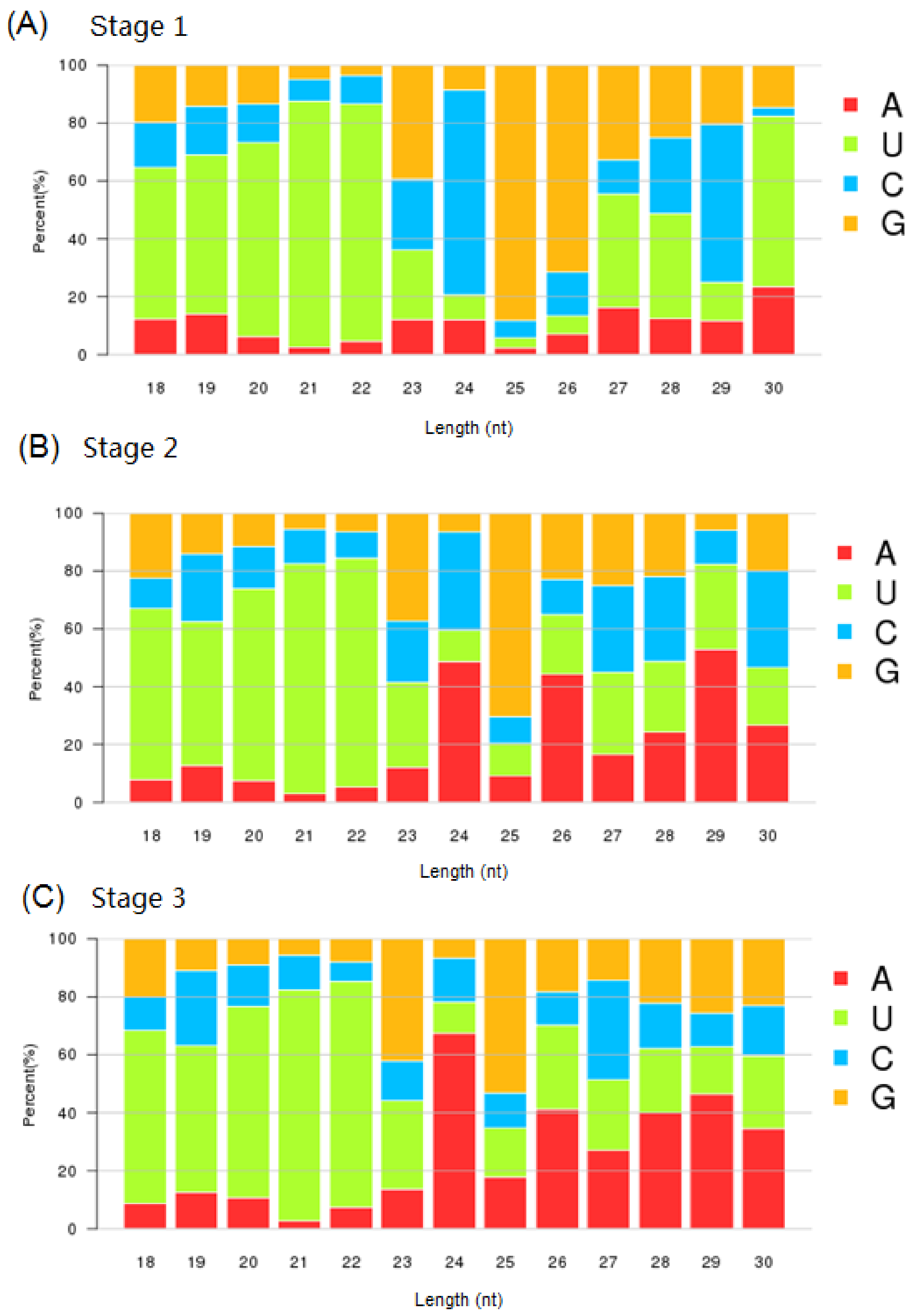

FIGURE S1 Base bias of miRNAs in *T. edulis*. (A) *T. edulis* stolon formation at the initial stage; (B) *T. edulis* stolon formation at the middle stage; (C) *T. edulis* stolon formation at the later stage.
